# Prediction of Mortality Risk After Ischemic Acute Kidney Injury With a Novel Prognostic Model: A Multivariable Prediction Model Development and Validation Study

**DOI:** 10.3389/fmed.2022.892473

**Published:** 2022-08-15

**Authors:** Mei Wang, Ping Yan, Ning-Ya Zhang, Ying-Hao Deng, Xiao-Qin Luo, Xiu-Fen Wang, Shao-Bin Duan

**Affiliations:** ^1^Department of Nephrology, Hunan Key Laboratory of Kidney Disease and Blood Purification, The Second Xiangya Hospital of Central South University, Changsha, China; ^2^Information Center, The Second Xiangya Hospital of Central South University, Changsha, China

**Keywords:** ischemia, acute kidney injury, mortality, risk factor, prognostic model

## Abstract

**Background and Objectives:**

Acute kidney injury (AKI) that results from ischemia is a common clinical syndrome and correlates with high morbidity and mortality among hospitalized patients. However, a clinical tool to predict mortality risk of ischemic AKI is not available. In this study, we aimed to develop and validate models to predict the 30-day and 1-year mortality risk of hospitalized patients with ischemic AKI.

**Methods:**

A total of 1,836 admissions with ischemic AKI were recruited from 277,898 inpatients admitted to three affiliated tertiary general hospitals of Central South University in China between January 2015 and December 2015. Patients in the final analysis were followed up for 1 year. Study patients were randomly divided in a 7:3 ratio to form the training cohort and validation cohort. Multivariable regression analyses were used for developing mortality prediction models.

**Results:**

Hepatorenal syndrome, shock, central nervous system failure, Charlson comorbidity index (≥2 points), mechanical ventilation, renal function at discharge were independent risk factors for 30-day mortality after ischemic AKI, while malignancy, sepsis, heart failure, liver failure, Charlson comorbidity index (≥2 points), mechanical ventilation, and renal function at discharge were predictors for 1-year mortality. The area under the receiver operating characteristic curves (AUROCs) of 30-day prediction model were 0.878 (95% confidence interval (CI): 0.849-0.908) in the training cohort and 0.867 (95% CI: 0.820–0.913) in the validation cohort. The AUROCs of the 1-year mortality prediction in the training and validation cohort were 0.803 (95% CI: 0.772–0.834) and 0.788 (95% CI: 0.741–0.835), respectively.

**Conclusion:**

Our easily applied prediction models can effectively identify individuals at high mortality risk within 30 days or 1 year in hospitalized patients with ischemic AKI. It can guide the optimal clinical management to minimize mortality after an episode of ischemic AKI.

## Introduction

Acute kidney injury (AKI) involved with various etiologies is a frequent clinical event among hospitalized patients. It is associated with increased length of hospital stay, development of chronic kidney disease, and high risk of mortality (1–4). The incidence of AKI has increased globally, which seriously endangers human health, making it a public health problem (2, 5, 6). As the result of ischemia, acute kidney injury is a common clinical syndrome characterized by low-perfusion states, occurring in a variety of clinical settings like hypovolemia, sepsis, multiple organ dysfunction, elective procedures, receiving therapies (7). In hospitalized patients the overall incidence of AKI is 11.6–23.9% (8, 9), and the mortality rate of AKI patients is around 10–20% (10, 11). When AKI occurred in COVID-19 positive patients, the proportion of death could be as high as 50% (12). Not surprisingly, some reports showed that prerenal AKI [one form of ischemic AKI (13)] accounted for more than half of the hospitalized AKI patients (14–16).

Existing evidence has emphasized comprehensive assessment of hospitalized admissions by making full use of clinical parameters to provide timely and precise predictions for detecting adverse outcomes of AKI (17). To a certain degree, some prognosis checklists could be used to assist in evaluation of the prognosis of ischemic AKI, especially in an intensive care unit (ICU). The most commonly used are Acute Physiology and Chronic Health Evaluation II (APACHE II) (18), and Sequential Organ Failure Assessment (SOFA) (19) prediction systems, but they have limited applicability for patients outside the ICU. In 2021 our team developed a successful mortality risk model in elderly hospitalized AKI patients. We found that 68.6% of AKI patients suffered from a renal ischemic injury and was associated with 19.4 and 30.2% of the 30-day and 1-year mortality, respectively (14). Under such serious circumstances, a timely and efficiently prognostic assessment tool of ischemic AKI is urgently demanding. Herein, we aimed to develop and validate risk prediction models for short-term (within 30 days) and long-term death (within 1 year) after an episode of ischemic AKI that can be calculated from readily available routine clinical variables, in the hope of improving clinical outcome of ischemic AKI.

## Patients and Methods

### Study Design and Population

We implemented a retrospective cohort study that included 277,898 inpatients of three affiliated tertiary general hospitals of Central South University in China from January 2015 to December 2015. There were 103,177 adult patients from the First Xiangya Hospital, 120,090 adult patients from the Second Xiangya Hospital, and 54,631 adult patients from the Third Xiangya Hospital. We selected patients who had at least two serum creatinine assays within any 7-day window during their first 30 days of hospitalization in the cohort. We used KDIGO criteria for the AKI definition and severity grading system (20). Until now, there are no consensus diagnostic criteria of ischemic AKI due to multiple etiologies and complex pathophysiology, and the condition is generally described as renal hypoperfusion (7). Therefore, patients with renal hypoperfusion and AKI were selected and classified as ischemic AKI. The exclusion criteria of participants were: (1) CKD stage 5 (chronic kidney disease patients with eGFR <15 ml/min/1.73 m^2^) or requiring long-term renal replacement therapy (dialysis or renal transplantation) before the hospital admission, (2) SCr change not attributed to AKI (e.g., SCr decrease after amputation), (3) hospital stay <48 h or incomplete medical records, (4) follow-up <1 year or loss. After exclusion, A total of 1,836 patients were selected in the final analysis. Eligible patients in the final analyses were followed up for 1 year after AKI diagnosis. For patients with multiple hospitalizations, we included only the first hospitalization. The manuscript reports result according to Strengthening the Reports of Observational Studies in Epidemiology (STROBE) guidelines (21).

### Statement of Ethics

The Medical Ethics Committee of the Second Xiangya Hospital of Central South University granted approval for the study protocol. This project has been registered by the Chinese Clinical Trial Registry (ChiCTR 1800019857, Registration Data: 12/2/2018). All the study methods were carried out according to the ethical standards of the Declaration of Helsinki. For this retrospective study, informed consent is deemed unnecessary.

### Data Collection and Definition

We collected the following patient-level data through electronic medical information system from participating hospitals. Baseline characteristics include demographics (sex and age), AKI stage, AKI type, ward of hospital admission, factors of ischemic injury, comorbidities, multiple organ failure, clinical procedure (22) (surgery, mechanical ventilation, dialysis, intravenous contrast use), laboratory test, renal recovery at discharge. In particularly, Community-acquired AKI (CA-AKI) was identified when patients met the KDIGO AKI definition according to serum creatinine change on the first day of admission, or the SCr was ≥ 1.4 mg/dl in men or ≥ 1.1 mg/dl in women on the first day of admission and≥1.5-fold of the minimal SCr level during hospitalization. Patients who developed AKI but did not meet community-acquired AKI criteria were classified as having Hospital-acquired AKI (HA-AKI) (8). Factors of ischemic injury were divided into hypovolemia (23), cardio-renal syndrome (24), hepatorenal syndrome (25) and others (7) accordance with our previous study (26) and relevant literature review (4, 7, 27). Information on significant comorbidities consisted of hypertension, diabetes, myocardial infarction, cerebrovascular disease, malignancy, pre-existing CKD (defined as eGFR < 60 ml/min/1.73 m^2^ predates the diagnosis of AKI), sepsis (28), shock, multiple organ failure (heart failure, respiratory failure, liver failure, central nervous system failure). These diseases were identified by the diagnosis codes at admission or discharge. In detail, the shock was defined as the systolic arterial pressure <90 mmHg or the mean arterial pressure <70 mmHg (29); heart failure included New York Heart Association class I–IV grades (30); respiratory failure referred to hypoxemia with oxygen saturation <60 mmHg (31). central nervous system failure referred to encephalopathy with Glasgow coma scale < 13 points without sedation (32). The burden of comorbidity was assessed using the Charlson comorbidity score simplified as CCI (< 2 vs. ≥ 2 points) (33). The laboratory test included baseline SCr, proteinuria (defined as dipstick urinalysis protein positive), metabolic acidosis (defined by a primary reduction in serum bicarbonate [$\text{HC}{\text{O}}_{3}^{-}$] concentration, a secondary decrease in the arterial partial pressure of carbon dioxide [*PaCO*_2_], and a reduction in blood potential of hydrogen (pH) (34), hypoalbuminemia (defined as serum albumin < 30 g/l). Hyperkalemia (serum K+ peak value > 5.5 mmol/l). Renal function recovery was assessed when the patients were discharged from the hospital and classified into three levels based on discharge SCr change: (1) Complete renal recovery at discharge was defined as full recovery with SCr the decrease to the below baseline or the physiological range. (2) Partial renal recovery was defined as the SCr decreased by 25% or higher from peak concentration but remaining higher than baseline or the physiological range and without the need for renal replacement therapy at discharge. (3) Failure renal recovery was defined as the decrease of SCr by <25% of peak concentration and the levels of SCr or patients still dependent on renal replacement therapy (16). The variables in the cohort as candidate predictors to develop mortality models of ischemic AKI.

### Identification of Ischemic AKI and Baseline SCr Definition

AKI is defined as an increase in SCr by > 0.3 mg/dl (26.5 μmol/l) from baseline within 48 h period or an increase in SCr ≥ 1.5 times baseline SCr within the prior 7 days being staged into three levels, with higher stages indicating greater severity. AKI stage 1 was defined as an increase in SCr by > 0.3 mg/dl (26.5 μmol/l) or 1.5–1.9 times baseline. Stage 2 as 2.0–2.9 times baseline. Stage 3 was more than 3.0 increase in SCr from the baseline or ≥ 4.0 mg/dl (353.6 μmol/l) or the initiation of RRT or in patients < 18 years in eGFR <35 ml/min/1.73m^2^ according to (KDIGO) Clinical Practice Guideline for AKI SCr criteria (20). Based on AKI definition, the patients with ischemic AKI are identified based on clinical adjudication of renal hypoperfusion. In detail, ischemic AKI patients were reviewed on a case-by-case basis to confirm the diagnosis with combined medical records, laboratory exams, related medical history by trained nephrologists. The lowest serum creatinine concentration was defined as the baseline creatinine with a previous serum creatinine value between 7 and 365 days before admission (35). If patients have no reliable records of baseline kidney function and without a history of chronic kidney disease, a back-estimation of the baseline SCr was obtained based on the equation of 4-variable Modification of Diet in Renal Disease (MDRD) formula with the assumption of an eGFR of 75 ml/min/1.73 m^2^ (36).

### Outcome Data

The primary clinical outcome was death from any cause within 30 days or 1 year after the diagnosis of ischemic AKI. The survival status and follow-up procedure were determined through reviewing all the relevant medical records (Hospital Information System, Laboratory Information System, and out-patient records), making phone calls, and sending a text message, and through data linkage to the Chinese Center for Disease Control and Prevention, which included almost complete coverage of death.

### Statistical Analysis

All the data collection and analysis were performed using SPSS software, version 26.0(IBM), and R software, version 4.1.2. Patients were divided into the training cohort (*n* = 1,282) and the validation cohort (*n* = 554) in a ratio of 7:3 through random sampling from the ischemic AKI patients. The training cohort was used for model construction and validation cohort was for validation. All descriptive statistics were summarized, and continuous data were expressed as medians with standard deviation. Categorical data were displayed as counts and percentages. Students *t*-test was used to compare the normal distribution continuous variables. The chi-square test was performed to compare categorical variables at baseline between each cohort. In the training cohort, univariable analysis was compared between groups stratified by survival status to explore potential predictors within 30-day and 1-year mortality. We included significant (P <0.05) predictors from univariable analysis and entered into multivariable-adjusted logistic-regression analysis with a stepwise backward elimination approach to simplify the models. Cumulative survival probabilities were assessed using a Kaplan-Meier time to event analysis with a log-rank test according to AKI classification and renal recovery at discharge. We evaluated the predictive accuracy of the model with discrimination and calibration, which was calculated with AUROC and Hosmer-Lemeshow test, respectively. The sensitivity and specificity, cutoff values were calculated by the AUROC analysis.

## Results

### Description of Cohort Baseline Characteristics

**Figure 1:**
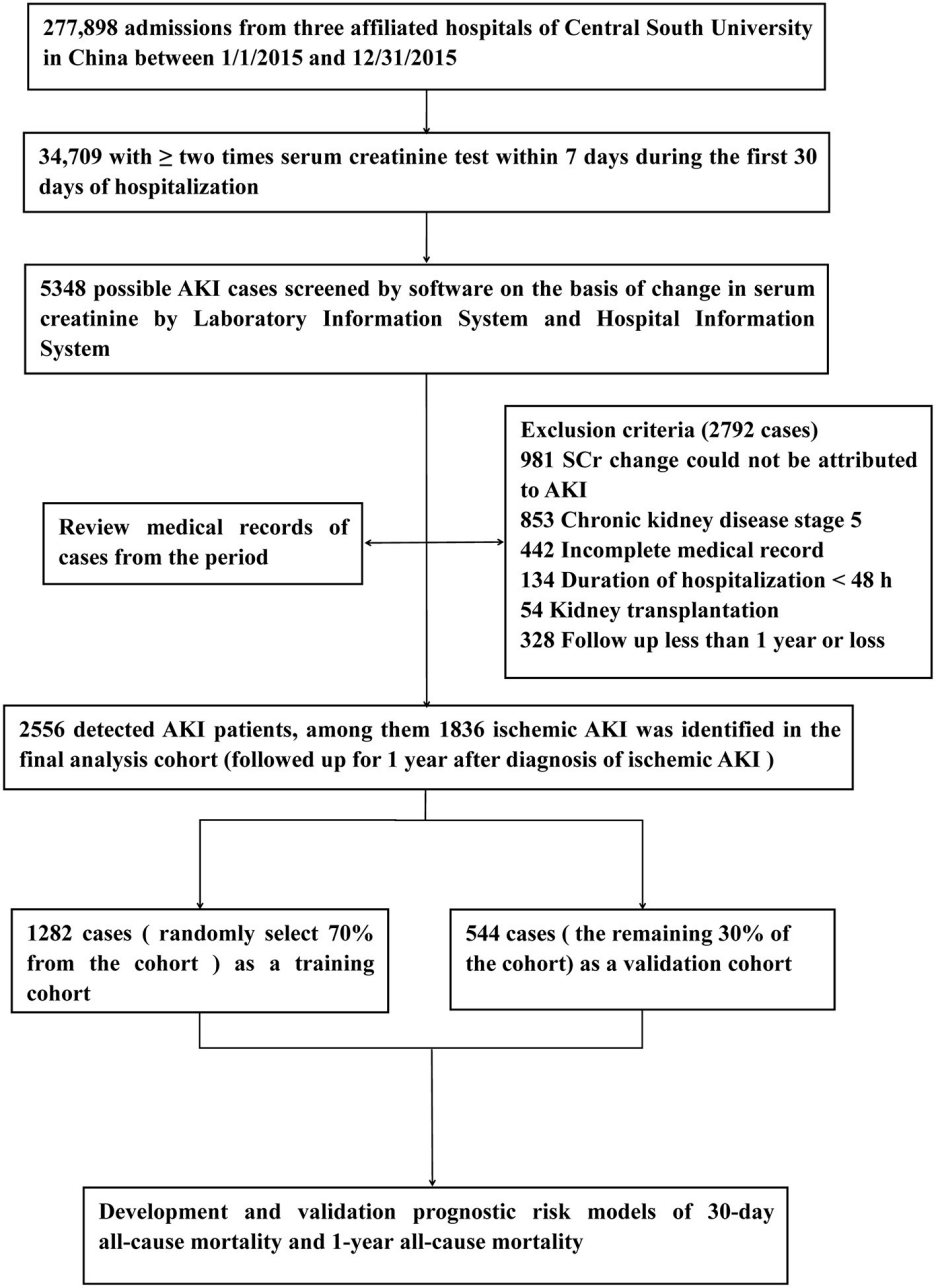
Study flow chart. AKI, Acute Kidney Injury.

We identified 1,836 patients with ischemic AKI between January 2015 to December 2015. A study flow chart detailing case selection and exclusion criteria have shown in Figure 1, in which the incidence of AKI hospitalized patients was 7.4% (2,556/34,709), and ischemic AKI occurred in 71.8% (1,836/2,556) of AKI individuals. As shown in Table 1, among the ischemic patients, more than half were male. About one third of patients were elder than 65 years old. The average age of the cohort was 55 years. There were 996 (54.2%) patients developed AKI stage 1, 397 (21.6%) developed AKI stage 2 and 443 (24.1%) developed AKI stage 3. Most patients were treated in the medical and surgical department with some treated in the intensive care unit. The training cohort included 1,282 patients, the remaining 554 as a validation cohort. Baseline characteristics of the training cohort and validation cohort were described in Table 1. The most common injury factor that might attribute to ischemia was hypovolemia. Nearly half of patients had recovered renal function by discharge in both cohorts. However, approximately 20% of patients having the severity of renal dysfunction are still dependent on renal replacement therapy or high levels of serum creatine at discharge. Except for other injury factors of ischemic AKI, there was no statistically significant differences between cohorts.

**Table 1:** Characteristics of the hospitalized ischemic patients in the training and validation cohort.

**Variable**	**Total (*n =* 1,836)**	**Cohort**		***P* Value**	**OR**	**95% CI**
		**Training cohort**	**Validation cohort**			
		**(*n =* 1,282)**	**(*n =* 554)**			
Age (M ± SD), years	55.18 ± 16.19	55.12 ± 16.10	55.33 ± 16.39	0.794	/	/
Age group, No. (%)				0.448	1.088	(0.875–1.352)
<65 years	1,273 (69.3)	882 (68.8)	391 (70.6)			
≥ 65 years	563 (30.7)	400 (31.2)	163 (29.4)			
Male, No. (%)	1,187 (64.7)	827 (64.5)	360 (65.0)	0.846	0.979	(0.795–1.207)
AKI stage, No. (%)				0.587		
Stage 1	996 (54.2)	705 (55.0)	291 (52.5)		/	/
Stage 2	397 (21.6)	275 (21.5)	122 (22.0)		0.930	(0.722–1.198)
Stage 3	443 (24.1)	302 (23.6)	141 (25.5)		0.884	(0.694–1.126)
AKI type, No. (%)				0.907	1.013	(0.821–1.248)
CA-AKI	636 (34.6)	443 (34.6))	193 (34.8)			
HA-AKI	1,200 (65.4)	839 (65.4)	361 (65.2)			
Ward of hospital admission, No. (%)				0.246		
Intensive-care unit	193 (10.5)	127 (9.9)	66 (11.9)		/	/
Medical	604 (32.9)	415 (32.4)	189 (34.1)		1.141	(0.809–1.609)
Surgical	1039 (56.6)	740 (57.7)	299 (54.0)		1.286	(0.928–1.782)
**Factors of ischemic injury, No. (%)**						
Hypovolemia	1507 (82.1)	1063 (82.9)	444 (80.1)	0.155	1.203	(0.932–1.551)
Cardiorenal syndrome	53 (2.9)	33 (2.6)	20 (3.6)	0.244	0.705	(0.401–1.241)
Hepatorenal syndrome	57 (3.1)	42 (3.3)	15 (2.7)	0.519	1.217	(0.669–2.214)
others	237 (12.9)	152 (11.9)	85 (15.3)	0.041	0.742	(0.557–0.988)
**Comorbidities, No. (%)**						
Hypertension	629 (34.3)	430 (35.5)	199 (35.9)	0.324	0.900	(0.731–1.109)
Diabetes	355 (19.3)	245 (19.1)	110 (19.9)	0.711	0.954	(0.742–1.226)
Myocardial infarction	86 (4.7)	52 (4.1)	34 (6.1)	0.053	0.647	(0.415–1.008)
Cerebrovascular disease	264 (14.4)	190 (14.8)	74 (13.4)	0.412	1.129	(0.845–1.507)
Malignancy	433 (23.6)	303 (23.6)	130 (23.5)	0.930	1.009	(0.798–1.277)
Pre-existing CKD	303 (16.5)	210 (16.4)	93 (16.8)	0.830	0.971	(0.743–1.269)
Sepsis	248 (13.5)	171 (13.3)	77 (13.9)	0.747	0.953	(0.714–1.274)
Shock	356 (19.4)	241 (18.8)	115 (20.8)	0.330	0.884	(0.689–1.133)
**Multiple organ failure, No. (%)**						
Heart failure	419 (22.8)	283 (22.1)	136 (24.5)	0.246	0.871	(0.689–1.100)
Respiratory failure	282 (15.4)	194 (15.1)	88 (15.9)	0.682	0.944	(0.718–1.242)
Liver failure	635 (34.6)	435 (33.9)	200 (36.1)	0.370	0.909	(0.738–1.120)
Central nervous system failure	375 (20.4)	266 (20.7)	109 (19.7)	0.600	1.069	(0.833–1.371)
CCI (≥2 points)	1,388 (75.6)	963 (75.1)	425 (76.7)	0.464	0.916	(0.725–1.158)
**Clinical Procedures, No. (%)**						
Surgery	886 (48.3)	625 (48.8)	261 (47.1)	0.519	1.068	(0.875–1.304)
Mechanical ventilation	447 (24.3)	312 (24.3)	135 (24.4)	0.989	0.998	(0.791–1.259)
Dialysis	221 (12.0)	150 (11.7)	71 (12.8)	0.500	0.901	(0.667–1.219)
Intravenous contrast use	332 (18.1%)	231 (18.0)	101 (18.2)	0.914	0.986	(0.761–1.277)
**Laboratory data**						
Baseline SCr (μmol/L)	76.70 ± 24.62	76.41 ± 24.69	77.37 ± 24.45	0.446	/	/
Proteinuria, No. (%)	235 (12.8)	169 (13.2)	66 (11.9)	0.455	1.123	(0.829–1.521)
Acidosis, No. (%)	576 (31.4)	397 (31.0)	179 (32.3)	0.569	0.940	(0.759–1.164)
Hypoalbuminemia, No. (%)	521 (28.4)	349 (27.2)	172 (31.0)	0.095	0.831	(0.668–1.033)
Hyperkalemia, No. (%)	175 (9.5)	121 (9.4)	54 (9.7)	0.836	0.965	(0.689–1.352)
Renal recovery at discharge, No. (%)				0.153		
Complete recovery	908 (49.5)	649 (50.6)	259 (46.8)		/	/
Partial recovery	573 (31.2)	399 (31.1)	174 (31.4)		0.915	(0.728–1.151)
Failed recovery	355 (19.3)	234 (18.3)	121 (21.8)		0.772	(0.594–1.003)

*AKI, Acute Kidney Injury; CA-AKI, Community Acquired AKI; HA-AKI, Hospital Acquired AKI; CKD, Chronic Kidney Disease; CCI, Charlson comorbidity index; SCr, Serum Creatinine.*

*Continuous variables were compared using Students t-test and categorical variables were compared using chi-square test*.

*A total of 1,836 patients included*.

### Clinical Outcomes Analysis

**Figure 2:**
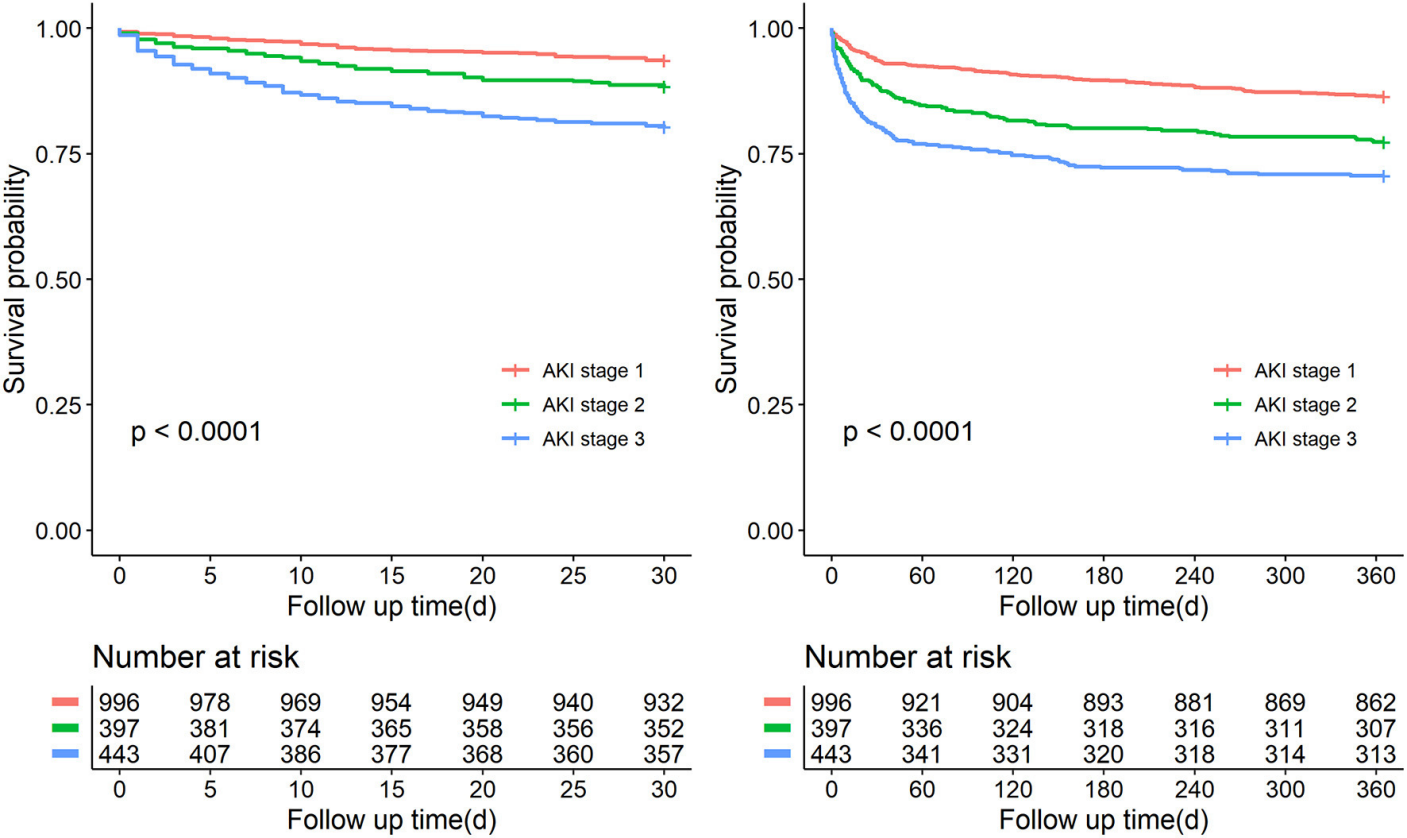
Patient survival probability within 30 days and 1 year of patients with different stages of AKI.

**Figure 3:**
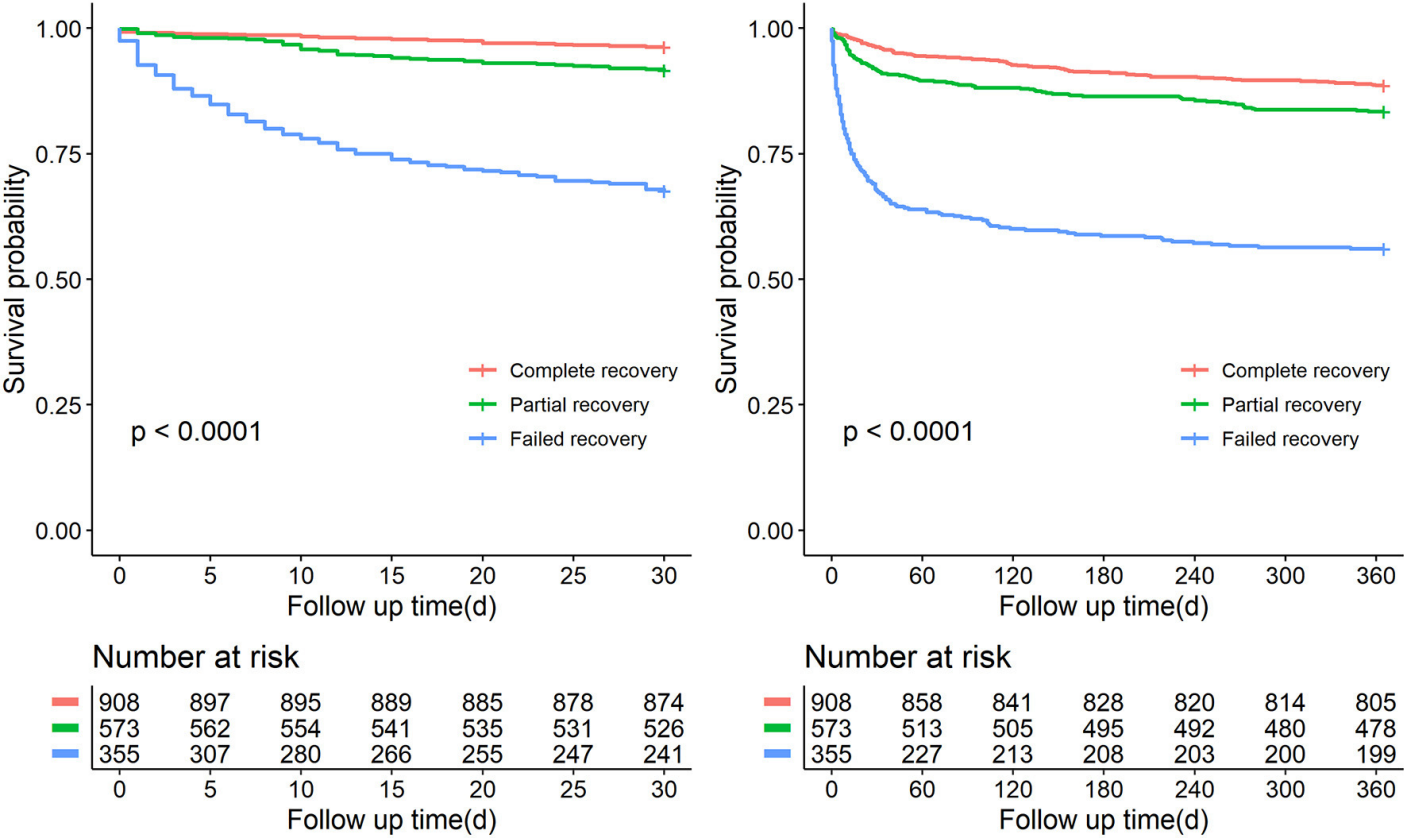
Patient survival probability within 30 days and 1 year of patients at discharge having different renal function status.

Over the observational course of the research in the training cohort, 136 (10.6%) patients passed away within 30-day, 250 (19.5%) ischemic AKI patients died within 1 year. In the validation cohort, the mortality of 30-day or 1-year was 11.0%, 19.0%, respectively. As shown in Figures 2, 3, we compared the overall survival probability of ischemic AKI patients according to AKI stages and renal recoveries separately without adjustment. The survival rate was significantly higher in the complete renal recovery and lower stage AKI individuals within 30 days and 1 year. However, no significance was found in the survival rate with different stages of AKI after adjusting for the covariate, suggesting that the severity of AKI was a cumulative risk factor of death rather an independent mortality predictor.

### Prediction of 30-Day All-Cause Mortality in the Patients With Ischemic AKI (Model 1)

As shown in Table 2, in the univariable analysis of the training cohort, we found that age, AKI stage, ward of hospital admission, hypovolemia, hepatorenal syndrome, myocardial infarction, sepsis, shock, multiple organ failure, Charlson comorbidity index (≥2 points), surgery, mechanical ventilation, dialysis, baseline SCr, acidosis, hypoalbuminemia, hyperkalemia, renal function at discharge in the training cohort were associated with death within 30-day. These parameters were entered into a multivariable logistic regression; we noted a significant association of 30-day death with several predictors that were studied previously: Hepatorenal syndrome, shock, central nervous system failure, Charlson comorbidity index (≥2 points), mechanical ventilation, renal function at discharge, which was quantified with the corresponding integrals of OR after adjusted confounding factors. For visualization, we developed an inter-based estimation system. The scoring criteria are displayed in Table 3.

**Table 2:** Characteristics of the study population stratified by 30-day survival status in the training cohort.

**Variable**	**Group**		***P* value**	**OR**	**95% CI**
	**Survival group (*n =* 1,146)**	**Death group (*n =* 136)**			
Age (M ± SD), years	54.70 ± 16.10	58.61 ± 15.81	0.007	/	/
Age group, No. (%)			0.139	1.322	(0.913–1.915)
<65 years	796 (69.5)	86 (63.2)			
≥ 65 years	350 (30.5)	50 (38.8)			
Male, No. (%)	734 (64.0)	93 (68.4)	0.318	1.214	(0.829–1.777)
AKI stage, No. (%)			<0.001		
Stage 1	659 (57.5)	46 (33.8)		/	/
Stage 2	240 (20.9)	35 (25.7)		2.089	(1.314–3.322)
Stage 3	247 (21.6)	55 (40.4)		3.190	(2.100–4.845)
AKI type, No. (%)			0.057	1.468	(0.987–2.183)
CA-AKI	406 (35.4)	37 (27.2)			
HA-AKI	740 (64.6)	99 (72.8)			
Ward of hospital admission, No. (%)			<0.001		
Intensive-care unit	97 (8.5)	30 (22.1)		/	/
Medical	363 (31.7)	52 (38.2)		0.463	(0.280–0.765)
Surgical	686 (59.9)	54 (39.7)		0.255	(0.155–0.417)
**Factors of ischemic injury, No. (%)**					
Hypovolemia	968 (84.5)	95 (69.9)	<0.001	0.426	(0.286–0.635)
Cardiorenal syndrome	29 (2.5)	4 (2.9)	0.775	1.167	(0.404–3.372)
Hepatorenal syndrome	28 (2.4)	14 (10.3)	<0.001	4.582	(2.349–8.938)
**Comorbidities, No. (%)**					
Hypertension	376 (32.8)	54 (39.7)	0.107	1.349	(0.936–1.943)
Diabetes	216 (18.8)	29 (21.3)	0.488	1.167	(0.754–1.805)
Myocardial infarction	41 (3.6)	11 (8.1)	0.012	2.372	(1.189–4.732)
Cerebrovascular disease	163 (14.2)	27 (19.9)	0.081	1.494	(0.950–2.349)
Malignancy	263 (22.9)	40 (29.4)	0.094	1.399	(0.944–2.074)
Pre-existing CKD	194 (16.9)	16 (11.8)	0.124	0.654	(0.380–1.127)
Sepsis	125 (10.9)	46 (33.8)	<0.001	4.175	(2.796–6.233)
Shock	166 (14.5)	75 (55.1)	<0.001	7.259	(4.985–10.570)
**Multiple organ failure, No. (%)**					
Heart failure	228 (19.9)	55 (40.4)	<0.001	2.734	(1.885–3.965)
Respiratory failure	117 (10.2)	77 (56.6)	<0.001	11.478	(7.777–16.941)
Liver failure	362 (31.6)	73 (53.7)	<0.001	2.510	(1.752–3.595)
Central nervous system failure	187 (16.3)	79 (58.1)	<0.001	7.108	(4.885–10.341)
CCI (≥2 points)	845 (73.7)	118 (86.8)	0.001	2.335	(1.398–3.901)
**Clinical Procedures, No. (%)**					
Surgery	581 (50.7)	44 (32.4)	<0.001	0.465	(0.319–0.678)
Mechanical ventilation	223 (19.5)	89 (65.4)	<0.001	7.838	(5.347–11.489)
Dialysis	112 (9.8)	38 (27.9)	<0.001	3.580	(2.347–5.461)
Intravenous contrast use	211 (18.4)	20 (14.7)	0.288	0.764	(0.465–1.256)
**Laboratory data**					
Baseline SCr (μmol/L)	75.44 ± 24.57	84.65 ± 24.31	<0.001	/	/
Proteinuria, No. (%)	150 (13.1)	19 (14.0)	0.774	1.078	(0.645–1.803)
Acidosis, No. (%)	323 (28.2)	74 (54.4)	<0.001	3.041	(2.119–4.364)
Hypoalbuminemia, No. (%)	296 (25.8)	53 (39.0)	0.001	1.834	(1.268–2.652)
Hyperkalemia, No. (%)	94 (8.2)	27 (19.9)	<0.001	2.772	(1.731–4.441)
Renal recovery at discharge, No. (%)			<0.001		
Complete recovery	624 (54.5)	25 (18.4)		/	/
Partial recovery	366 (31.9)	33 (24.3)		2.250	(1.317–3.844)
Failed recovery	156 (13.6)	78 (57.4)		12.480	(7.696–20.238)

*AKI, Acute Kidney Injury; CA-AKI, Community Acquired AKI; HA-AKI, Hospital Acquired AKI; CKD, Chronic Kidney Disease; CCI, Charlson comorbidity index; SCr, Serum Creatinine*.

*Continuous variables were compared using Students t-test and categorical variables were compared using chi-square test*.

*A total of 1,282 patients included*.

**Table 3:** Predictors for 30-day mortality in the hospitalized ischemic AKI patients.

**Variables**	***P* value**	**OR (95%CI)**	**Categories**	**Score**
Hepatorenal syndrome	0.002	3.671 (1.633–8.254)	No	0
			Yes	4
Shock	0.002	2.136 (1.322–3.450)	No	0
			Yes	2
Central nervous system failure	0.001	2.185 (1.353–3.526)	No	0
			Yes	2
CCI (≥2 points)	0.017	2.025 (1.132–3.625)	No	0
			Yes	2
Mechanical ventilation	<0.001	4.465 (2.703–7.377)	No	0
			Yes	4
Renal recovery at discharge	-	1.000 (Reference)	Complete recovery	0
	0.001	2.563 (1.448–4.535)	Partial recovery	3
	<0.001	6.930 (4.057–11.839)	Failed recovery	7
Maximum points				21

*Six-variable risk index formula for affecting 30-day mortality in the hospitalized ischemic AKI patients*.

*CCI, Charlson comorbidity index; CI, confidence interval; OR, odds ratio*.

*The predictors were analyzed by multivariable logistic regression*.

*A total of 1,282 patients included*.

### Validation and Evaluation of Model 1 in Hospitalized Ischemic AKI Patients

As shown in Table 4, model 1 was well-calibrated for 30-day mortality prediction with the overall goodness-of-fit (*P* = 0.091) using Hosmer-Lemeshow (H-L) test. AUROC was 0.878 (95% CI: 0.849–0.908), and the model achieved a sensitivity of 0.86 and specificity of 0.78 in the training cohort. In the validation cohort, the AUROC was 0.867 (95% CI: 0.820–0.913), and the sensitivity and specificity were 0.82 and 0.76. The cutoff value was 8, which was determined based on the best Youden index of 0.640. All the patients were divided into two risk stratification (low-risk group: 0–7 points vs. high-risk group: ≥ 8 points) based on the cutoff value. The classification cross table for observed outcome and predicted outcome for 30-day mortality was provided in Supplementary Table S1. The results indicated model 1 was reliable in predicting the all-cause death within 30 days.

**Table 4:** Predictive performance of prediction model system for ischemic AKI within 30-day and 1-year all-cause death in the training cohort and validation cohort.

	**Goodness-of-fit (*P* value)**	**AUROC ±SD (95%CI)**	**Sensitivity (%)**	**Specificity (%)**	**Cut off point**	**Youden index**
**Model 1**
Training cohort (*n =* 1,282)	8.016 (0.091)	0.878 ± 0.015 (0.849–0.908)	0.86	0.78	8	0.640
Validation cohort (*n =* 554)	_	0.867 ± 0.024 (0.820–0.913)	0.82	0.76	8	0.577
**Model 2**
Training cohort (*n =* 1,282)	9.597 (0.213)	0.803 ± 0.016 (0.772–0.834)	0.75	0.73	7	0.482
Validation cohort (*n =* 554)	_	0.788 ± 0.024 (0.741–0.835)	0.73	0.68	7	0.417

*AUROC, the area under the receiver operating characteristic curves; DS, standard deviation; CI, confidence interval*.

*The models' performance was analyzed by Hosmer-Lemeshow (H-L) test and ROC analysis*.

*A total of 1,836 patients included*.

### Prediction of 1-Year All-Cause Mortality in the Patients With Ischemic AKI (Model 2)

As shown in Table 5 age, gender, AKI stage, AKI type, ward of hospital admission, hypovolemia, hepatorenal syndrome, diabetes, cerebrovascular disease, malignancy, pre-existing CKD, sepsis, shock, multiple organ failure, Charlson comorbidity index (≥2 points), surgery, mechanical ventilation, dialysis, baseline SCr, acidosis, hypoalbuminemia, hyperkalemia, renal function at discharge in the training cohort were associated with 1-year mortality after univariable analysis. By multivariate logistic regression, seven variables including malignancy, sepsis, heart failure, liver failure, Charlson comorbidity index (≥2 points), mechanical ventilation, renal function at discharge were shown to be independently associated with death within 1 year. The corresponding integrals of various OR values of the independent risk factors were endowed, and each patients' score was calculated based on the prognostic risk formula of 1-year mortality in Table 6.

**Table 5:** Characteristics of the study population stratified by 1-year survival status in the training cohort.

**Variable**	**Group**		***P* value**	**OR**	**95% CI**
	**Survival group (*n =* 1,032)**	**Death group (*n =* 250)**			
Age (M ± SD),years	54.02 ± 15.98	59.62 ± 15.85	<0.001	/	/
Age group, No. (%)			<0.001	1.662	(1.248–2.212)
<65 years	733 (71.0)	149 (59.6)			
≥ 65 years	299 (29.0)	101 (40.4)			
Male, No. (%)	652 (63.2)	175 (70.0)	0.043	1.36	(1.009–1.833)
AKI stage, No. (%)			<0.001		
Stage 1	608 (58.9)	97 (38.8)		/	/
Stage 2	208 (20.2)	67 (26.8)		2.019	(1.424–2.862)
Stage 3	216 (20.9)	86 (34.4)		2.496	(1.796–3.469)
AKI type, No. (%)			0.015	1.455	(1.074–1.972)
CA-AKI	373 (36.1)	70 (28.0)			
HA-AKI	659 (63.9)	180 (72.0)			
Ward of hospital admission, No. (%)			<0.001		
Intensive-care unit	86 (8.3)	41 (16.4)		/	/
Medical	318 (30.8)	97 (38.8)		0.64	(0.414–0.989)
Surgical	628 (60.9)	112 (44.8)		0.374	(0.245–0.571)
**Factors of ischemic injury, No. (%)**					
Hypovolemia	877 (85.0)	186 (74.4)	<0.001	0.514	(0.369–0.715)
Cardiorenal syndrome	23 (2.2)	10 (4.0)	0.113	1.828	(0.859–3.892)
Hepatorenal syndrome	24 (2.3)	18 (7.2)	<0.001	3.259	(1.740–6.104)
**Comorbidities, No. (%)**					
Hypertension	336 (32.6)	94 (37.6)	0.130	1.248	(0.937–1.663)
Diabetes	185 (17.9)	60 (24.0)	0.028	1.446	(1.038–2.013)
Myocardial infarction	40 (3.9)	12 (4.8)	0.506	1.25	(0.646–2.420)
Cerebrovascular disease	138 (13.4)	52 (20.8)	0.003	1.701	(1.194–2.424)
Malignancy	221 (21.4)	82 (32.8)	<0.001	1.791	(1.323–2.425)
Pre-existing CKD	180 (17.4)	30 (12.0)	0.037	0.645	(0.427–0.976)
Sepsis	104 (10.1)	67 (26.8)	<0.001	3.267	(2.312–4.615)
Shock	141 (13.7)	100 (40.0)	<0.001	4.213	(3.092–5.739)
**Multiple organ failure, No. (%)**					
Heart failure	188 (18.2)	95 (38.0)	<0.001	2.752	(2.038–3.715)
Respiratory failure	90 (8.7)	104 (41.6)	<0.001	7.456	(5.351–10.388)
Liver failure	314 (30.4)	121 (48.4)	<0.001	2.145	(1.619–2.841)
Central nervous system failure	150 (14.5)	116 (46.4)	<0.001	5.09	(3.760–6.891)
CCI (≥2 points)	742 (71.9)	221 (88.4)	<0.001	2.978	(1.976–4.489)
**Clinical Procedures, No. (%)**					
Surgery	537 (52.0)	88 (35.2)	<0.001	0.501	(0.376–0.667)
Mechanical ventilation	186 (18.0)	126 (50.4)	<0.001	4.622	(3.443–6.204)
Dialysis	94 (9.1)	56 (22.4)	<0.001	2.88	(1.999–4.150)
Intravenous contrast use	191 (18.5)	40 (16.0)	0.355	0.839	(0.578–1.218)
**Laboratory data**					
Baseline SCr (μmol/L)	75.40 ± 24.93	80.60 ± 23.26	0.003	/	/
Proteinuria, No. (%)	137 (13.3)	32 (12.8)	0.842	0.959	(0.635–1.448)
Acidosis, No. (%)	278 (26.9)	119 (47.6)	<0.001	2.464	(1.855–3.272)
Hypoalbuminemia, No. (%)	258 (25.0)	91 (36.4)	<0.001	1.717	(1.280–2.303)
Hyperkalemia, No. (%)	80 (7.8)	41 (16.4)	<0.001	2.334	(1.557–3.500)
Renal recovery at discharge, No. (%)			<0.001		
Complete recovery	569 (55.1)	80 (32.0)		/	/
Partial recovery	337 (32.7)	62 (24.8)		1.309	(0.915–1.872)
Failed recovery	126 (12.2)	108 (43.2)		6.096	(4.306–8.631)

*AKI, Acute Kidney Injury; CA-AKI, Community Acquired AKI; HA-AKI, Hospital Acquired AKI; CKD, Chronic Kidney Disease; CCI, Charlson comorbidity index; SCr, Serum Creatinine*.

*Continuous variables were compared using Students t-test and categorical variables were compared using chi-square test*.

*A total of 1,282 patients included*.

**Table 6:** Predictors for 1-year mortality in the hospitalized ischemic AKI patients.

**Variables**	***P* value**	**OR 95%CI**	**Categories**	**Score**
Malignancy	<0.001	2.767 (1.893–4.044)	No	0
			Yes	3
Sepsis	0.021	1.665 (1.081–2.564)	No	0
			Yes	2
Heart failure	<0.001	2.028 (1.402–2.935)	No	0
			Yes	2
Liver failure	0.002	1.657 (1.200–2.289)	No	0
			Yes	2
CCI (≥2 points)	0.001	2.239 (1.394–3.597)	No	0
			Yes	2
Mechanical ventilation	<0.001	3.514 (2.444–5.053)	No	0
			Yes	4
Renal recovery at discharge	-	1.000 (Reference)	Complete recovery	0
	0.181	1.298 (0.886–1.903)	Partial recovery	1
	<0.001	4.568 (3.112–6.706)	Failed recovery	5
Maximum points				20

*Seven-variable risk index formula for affecting 1-year mortality in the hospitalized ischemic AKI patients*.

*CCI, Charlson comorbidity index; CI, confidence interval; OR, odds ratio*.

*The predictors were analyzed by multivariable logistic regression*.

*A total of 1,282 patients included*.

### Validation and Evaluation of Model 2 in Hospitalized Ischemic AKI Patients

As shown in Table 4, model 2 showed good calibration with a high goodness-of-fit (0.213) assessing with the H-L test. The AUROC of the model 2 were 0.803 (95%CI: 0.772–0.834) in the training cohort and 0.788 (95%: CI:0.741–0.835) in the validation cohort. The sensitivity and specificity were 0.75 and 0.73 in the training cohort and 0.73 and 0.68 in the validation cohort. The cutoff value was set at 7. Risk stratification was conducted for all patients as a low-risk group (0–6 points) and high-risk group (≥ 7 points). We also performed an analysis for observed outcome and predicted outcome of 1-year mortality with a classification cross table in Supplementary Table S2, showing the good prediction effectiveness of prognostic model for 1-year mortality.

### Subgroup Analysis Between Medical and Surgical Patients

In our study, more than half of the patients were admitted in the surgical department. We also conducted a subgroup analysis between medical patients and surgical patients in Supplementary Table S3, the profiles of risk factors differed markedly between these two groups. Compared with medical patients, the surgical patients have several features, they were more younger and half of patients at the early stage of kidney dysfunction with relative higher frequency use of medical contrast. In medical department, patients were older relatively with more comorbidities. The mortality risk was significantly higher in medical patients. We also performed our models in surgical and medical patients. The model performance in the surgical and medical patients is shown in Supplementary Table S4. Model discrimination remained consistent in both subsets of patients.

## Discussion

Our study provided a comprehensive analysis of ischemic AKI among hospitalized patients. A large number of ischemic AKI patients were identified, we found ischemic AKI is a leading cause of overall AKI hospital admissions in our study. With the dynamic evolution of ischemic AKI, we found that Charlson comorbidity index (≥2 points), mechanical ventilation and renal recovery at discharge were common risk factors for 30-day and 1-year mortality. We further established two novel prognostic risk models to predict the all-cause death within 30 days and 1 year, respectively. The models provided a significant scientific theoretical basis for early identification of ischemic AKI prognosis and could give timely interventions for individuals at high risk of mortality.

Renal ischemia is a major clinical problem without effective therapy and is a significant cause of morbidity and mortality (37). Epidemiologic studies on ischemic AKI remained scarce. The most common factors contributed to ischemic AKI was hypovolemia with heterogeneities and often accompanied by multiple organ failure, sepsis (7). Ischemic AKI is frequently associated with patients undergoing surgery (38). These clinical characters showed high agreement with our study. Given the heterogeneous and multifactor etiology of ischemic AKI, the incidence estimates vary widely among different patient-related risk factors. The incidence of AKI in hospitalized patients is 11.6–23.9% (8, 9), while the incidence of ischemic AKI of overall AKI patients is range from 55.6 to 77.8% (14–16, 26), approximately. In our study, the incidence of ischemic AKI among hospitalized patients is 5.3% and 71.8% in overall AKI patients, demonstrating ischemic AKI common among hospitalized AKI patients. These findings were similar to a previous epidemiological investigation on AKI in China (16). This study revealed renal hypoperfusion as a most implicate cause accounting for 77.8% of all AKI patients originating from 44 study hospitals of 22 provinces in China. This survey adds strength to the estimate of ischemic AKI, which indicated our subsequent studies were rational. Our results showed the 30-day all-cause mortality rate of ischemic AKI was 11.7%, and 1-year was 19.3%. However, information on the prognosis of ischemic AKI in hospitalized patients is limited. Recently, a multicenter epidemiological cohort from Denmark covering 30,762 patients showed that the prevalence of AKI was 15.6% based on the RIFLE classification, the overall 30-day mortality was 39.6%, and 1-year was 52.9%. What's more, the risk of mortality increased with the severity of AKI, which was quite different from our results (39). Contributory reasons for differences were inconsistent diagnostic criteria for AKI and failed to analyze ischemic AKI outcomes independently. In our results, the severity of AKI was not an independent risk factor for adverse effects after adjusted covariables. A plausible explanation was that in our cohort, the patients experienced an acute ischemic decline in kidney function with more severe comorbidities, which indicated the mortality was not only caused by AKI but also underlying concomitant disease. The causes of death may be a combination of these factors. These may also interpret some critically ill patients who often develop AKI as a complication of the original disorder (40).

This study conducted two practical prognostic scoring systems: model 1 and model 2, to predict the mortality risk within 30-day and 1-year after ischemic AKI. Both models showed satisfying performance in predicting whether individuals would probably die within 30 days or 1 year. These two validated models showed good calibration and discrimination with relatively large AUROCs, reliable sensitivity, and specificity. Models are usually considered significant for clinical guidance when AUROC is >0.75. Compared with AKI mortality with our previous study, both models showing reliable prediction performance in 30-day mortality (sensitivity: 0.86 vs. 0.83, specificity: 0.78 vs. 86.0) and 1-year mortality (sensitivity: 0.75 vs. 0.77, specificity: 0.73 vs. 0.82). Wu et al. investigated association between the nadir platelet count and AKI or 28-day all-cause mortality after hemorrhagic shock. The platelet count predicted the 28-day cause mortality with AUROC of 0.76, and the sensitivity and specificity were 0.84 and 0.66, respectively. Suggesting our models outperformed in predicting death at 30 days (41). Our findings were further extended previous works in this area. Bonventre JV (37) revealed ischemic AKI was often associated with multiple organ failures and sepsis as well as carried a marked increase in mortality. Chertow G (42) provided prognostic stratification and risk adjustment models to predict mortality after AKI, which demonstrated a strong link between extra-renal organ system failures and the risk of death. This study only took AKI patients in critically ill into account and was not validated. Our previous studies conducted and validated mortality models for elderly AKI patients (14) and established scoring systems for AKI progression to AKD as well as 90-day mortality or end stage renal disease (26). However, these studies investigated all AKI mortality risks but failed to focus on ischemic AKI subgroup analysis. These studies also did not consider renal ischemia as an independent risk factor that could lead to worse outcomes. Herein, we emphasized the necessity for more attention to the outcome study of this group to minimize mortality as much as possible and get closer to the initiatives of the International Society of Nephrology AKF “0 by 25” (43).

This study has several strengths. As far as we know, we had first shown the incidence and mortality risk factors of ischemic AKI in hospitalized patients in China. We innovatively conducted two prognostic scoring systems for 30-day and 1-year death. These models were practical tools that could be used at the bedside, and they were used to evaluate the death risk of ischemic AKI. Consistent with KDIGO advocacy for follow-up of all AKI patients, in these two models, we divided patients into the high-risk and low-risk groups based on the cutoff value. Therefore, clinicians can identify high risk of patients to timely provide targeted therapy, avoid aggravation, and inform the adverse outcome. It is crucial for a nephrologist to guide survivors with individualized follow-up plans and management strategies based on the model's risk stratification to avoid unnecessary medical resources. Even though the pathologic mechanism and prognosis of ischemic AKI remain poorly understood, our analysis provided important scientific prognostic information for ischemic AKI. From a clinical perspective, our models could direct the management of AKI patients to avoid further insult, lower the mortality rate, reduce disease burden.

In addition, there are several limitations in our study. Firstly, we specifically focused on hypovolemia, cardiorenal syndrome, and hepatorenal syndrome associated with ischemic AKI and lack of a gold diagnostic standard for its definition, potentially making our results underestimate the burden of ischemic AKI. Second, the urine output criteria were not used to define AKI due to the lack of urinary data for most patients. But some scholars indicated urine output to identify AKI may be liberal (44). Third, the results might be affected by the possible selection bias due to the nature of retrospective study. Forth, this study represented a retrospective cohort in a single province, and some outcome data were obtained via phone calls or text messages, which may lead to sampling bias and recall bias. The models are constructed and validated internally. It is therefore uncertain whether it affects the generalization in other regions. It is necessary to conduct a multi-center prospective cohort to evaluate the accuracy of the models.

## Conclusion

In conclusion, we found ischemic AKI was the most common form of AKI. We explored risk factors of mortality within 30-day and 1-year furtherly established and validated two corresponding prognostic risk models using readily available routine clinical variables as an alarm system to identify individuals at high mortality risk. The models could be useful clinical tools to benefit high-risk patients, give personalized treatment plans, and improve outcome.

## Data Availability Statement

The datasets were analyzed during the current study available from the corresponding author on reasonable request.

## Ethics Statement

The studies involving human participants were reviewed and approved by the Medical Ethics Committee of the Second Xiangya Hospital of Central South University. Written informed consent for participation was not required for this study in accordance with the national legislation and the institutional requirements.

## Author Contributions

S-BD designed and supervised the study and drafted the manuscript. MW performed the data extraction, analyzed and interpreted the data, and drafted the manuscript. PY and N-YZ analyzed and interpreted the data and critically revised the manuscript. Y-HD, X-QL, and X-FW analyzed the data and revised the manuscript critically for important intellectual content. All authors read and approved the final manuscript.

## Funding

This study was supported by National Natural Science Foundation of China (Nos. 81873607 and 81570618), Development and Reform Commission of Hunan Province (2014-658), Scientific Foundation of Hunan Province, China (S2013F1022), and Clinical Medical Technology Innovation Guide Project of Hunan Province (2017SK50117).

## Conflict of Interest

The authors declare that the research was conducted in the absence of any commercial or financial relationships that could be construed as a potential conflict of interest.

## Publisher's Note

All claims expressed in this article are solely those of the authors and do not necessarily represent those of their affiliated organizations, or those of the publisher, the editors and the reviewers. Any product that may be evaluated in this article, or claim that may be made by its manufacturer, is not guaranteed or endorsed by the publisher.
