# Supplementary material for: Prediction of Mortality Risk After Ischemic Acute Kidney Injury With a Novel Prognostic Model: A Multivariable Prediction Model Development and Validation Study
Source: Front Med (Lausanne). 2022 Aug 15;9:892473. doi: 10.3389/fmed.2022.892473 (PMC9420861; doi:10.3389/fmed.2022.892473)
Supplement: Supplementary file 1 [file Data_Sheet_1.PDF]

Table S1. Classification cross table for the predicted outcome and observed outcome for 30-day mortality in ischemic AKI patients

model 1	observed outcome	
	death	survivor
death	157	317
survivor	40	1322

Sensitivity =157 / (157+40) * 100%= 79.70%
Specificity =1322 / (317+1322) * 100%= 80.66%

Table S2. Classification cross table for the predicted outcome and observed outcome for 1-year mortality in ischemic AKI patients

model 2	observed outcome	
	death	survivor
death	264	417
survivor	91	1064

Sensitivity =264 / (264+91) * 100%= 74.37%
Specificity=1064 / (417+1064) * 100%=71.84 %

Table S3. Characteristics between surgical and medical ischemic AKI patients

Variable	Medical patients (n=604)	Surgical patients (n=1039)	*P* Value	OR	95% CI
Age (M ± SD), years	55.89±17.95	53.99±14.61	＜0.001	/	/
**Age group, No. (%)**			＜0.001	0.619	(0.498-0.770)
< 65 years	389 (64.4)	744 (74.5)			
≥ 65 years	215 (35.6)	265 (25.5)			
Male, No. (%)	401 (66.4)	657 (63.2)	0.198	0.871	(0.705-1.075)
**AKI stage, No. (%)**			＜0.001		
Stage 1	249 (41.2)	660 (63.5)		/	/
Stage 2	158 (26.2)	204 (19.6)		0.487	(0.378-0.628)
Stage 3	197 (32.6)	175 (16.8)		0.335	(0.261-0.431)
**AKI type, No. (%)**			＜0.001	3.888	(3.128-4.832)
CA-AKI	312 (51.7)	224 (21.6)			
HA-AKI	292 (48.3)	815 (78.4)			
**Factors of ischemic injury, No. (%)**					
Hypovolemia	445 (73.7)	920 (88.5)	＜0.001	2.762	(2..123-3.594)
Cardiorenal syndrome	32 (5.3)	18 (1.7)	＜0.001	0.315	(0.175-0.566)
Hepatorenal syndrome	38 (6.3)	16 (1.5)	＜0.001	0.233	(0.129-0.422)
Others	97 (16.1)	93 (9.0)	＜0.001	0.514	(0.379-0.697)
**Comorbidities, No. (%)**					
Hypertension	241 (39.9)	304 (29.3)	＜0.001	0.623	(0.505-0.769)
Diabetes	145 (24.0)	154 (14.8)	＜0.001	0.551	(0.428-0.710)
Myocardial infarction	45 (7.5)	18 (1.7)	＜0.001	0.219	(0.126-0.382)
Cerebrovascular disease	84 (13.9)	124 (11.9)	0.246	0.839	(0.623-1.129)
Malignancy	86 (14.2)	332 (32.0)	＜0.001	2.828	(2.175-

					3.679)
Pre-existing CKD	173 (28.6)	101 (9.7)	＜0.001	0.268	(0.205-0.352)
Sepsis	83 (13.7)	87 (8.4)	0.001	0.574	(0.417-0.789)
Shock	119 (19.7)	155 (14.9)	0.012	0.715	(0.549-0.930)
**Multiple organ failure, No. (%)**					
Heart failure	193 (32.0)	161 (15.5)	＜0.001	0.390	(0.307-0.496)
Respiratory failure	95 (15.7)	103 (9.9)	＜0.001	0.590	(0.437-0.795)
Liver failure	190 (31.5)	353 (34.0)	0.295	1.121	(0.905-1.389)
Central nervous system failure	119 (19.7)	160 (15.4)	0.025	0.742	(0.571-0.964)
CCI (≥2 points)	483 (80.0)	573 (72.5)	＜0.001	0.660	(0.518-0.840)
**Clinical Procedures, No. (%)**					
Surgery	92 (15.2)	761 (73.2)	＜0.001	15.234	(11.735-19.778)
Mechanical ventilation	94 (15.6)	243 (23.4)	＜0.001	1.656	(1.274-2.154)
Dialysis	97 (16.1)	64 (6.2)	＜0.001	0.343	(0.246-0.479)
Intravenous contrast use	89 (14.7)	222 (21.4)	0.001	1.572	(1.202-2.059)
**Laboratory data**					
Baseline SCr (μmol/L)	81.97±26.94	73.93±22.81	0.199	/	/
Proteinuria, No. (%)	116 (19.2)	66 (6.4)	＜0.001	0.285	(0.207-0.393)
Acidosis, No. (%)	207 (34.3)	267 (25.7)	＜0.001	0.663	(0.533-0.825)
Hypoalbuminemia, No. (%)	235 (38.9)	209 (20.1)	＜0.001	0.395	(0.316-0.494)
Hyperkalemia, No. (%)	71 (11.8)	78 (7.5)	0.004	0.609	(0.434-0.855)
**Renal recovery at discharge, No. (%)**			＜0.001		
Complete recovery	280 (46.4)	548 (52.7)		/	/

Partial recovery	174 (28.8)	350 (33.7)		1.028	(0.815-1.296)
Failed recovery	150 (24.8)	141 (13.6)		0.480	(0.366-0.630)
**Death, No. (%)**					
Death within 30 days	77 (12.7)	75 (7.2)	＜0.001	0.532	(0.381-0.630)
Death within 1 year	141 (23.3)	152 (14.6)	＜0.001	0.563	(0.436-0.726)

AKI = Acute Kidney Injury; CA-AKI = Community Acquired AKI; HA-AKI = Hospital Acquired AKI; CKD = Chronic Kidney Disease; CCI = Charlson comorbidity index; SCr= Serum Creatinine.

Continuous variables were compared using Students t-test and categorical variables were compared using chi-square test. A total of 1643 patients included.

Table S4. Model performance for 30-day and 1-year mortality between surgical and medical patients

	AUROC ± SD (95%CI)	Sensitivity (%)	Specificity (%)	Cut off point	Youden index
**Model 1**					
Medical patients (n=604)	0.869 ± 0.023 (0.825-0.913)	0.84	0.76	8	0.603
Surgical patients (n=1039)	0.884 ± 0.019 (0.847-0.921)	0.79	0.82	8	0.609
**Model 2**					
Medical patients (n=604)	0.805 ± 0.021 (0.765-0.846)	0.70	0.74	7	0.434
Surgical patients (n=1039)	0.791 ± 0.020 (0.751-0.831)	0.72	0.74	7	0.466

AUROC = the area under the receiver operating characteristic curves; DS = standard deviation; CI = confidence interval.

The models' performance was analyzed by Hosmer-Lemeshow (H-L) test and ROC analysis.

A total of 1643 patients included.
